# Identifying early-life, environmental, and social stressors as key predictors of U.S. respiratory failure mortality: a machine learning study

**DOI:** 10.3389/fpubh.2026.1839753

**Published:** 2026-06-26

**Authors:** Changhua Yang, Wei Zhang, Jiewei Liu

**Affiliations:** 1 Department of Emergency Medicine, Fuzong Clinical Medical College of Fujian Medical University, Fuzhou, Fujian, China; 2 Department of Emergency, 900th Hospital of PLA Joint Logistic Support Force, Fuzhou, Fujian, China; 3 Department of Emergency Medicine, Fuzong Teaching Hospital of Fujian University of Traditional Chinese Medicine (900th Hospital), Fuzhou, Fujian, China

**Keywords:** CDC WONDER, explainable artificial intelligence, GBD, GBM, machine learning, mortality, public health, respiratory failure

## Abstract

**Background:**

Traditional risk factors like smoking and air pollution remain insufficient to explain the persistent geographical variation in respiratory failure mortality across the United States, with significantly higher mortality observed in specific regions such as the Mississippi River Basin.

**Methods:**

We conducted a state-level ecological study using Centers for Disease Control and Prevention Wide-ranging Online Data for Epidemiologic Research (CDC WONDER) mortality data and 68 socio-environmental risk factors from Global Burden of Disease (GBD). Predictive modeling leveraged 12 machine learning algorithms (from linear to tree-based ensembles) with Recursive Feature Elimination (RFE) and SHapley Additive exPlanations (SHAP) interpretation.

**Results:**

The optimal model Gradient Boosting Machine (GBM) achieved high accuracy (Root Mean Square Error (RMSE) = 0.7447, R-squared (R^2^) = 0.872), identifying four dominant state-level predictors: short gestation, residential radon, intimate partner violence, and occupational nickel exposure. Key interactions, such as nickel-radon ecological co-patterns, were uncovered.

**Conclusion:**

These findings suggest an “early-life and cumulative exposure” framework as a hypothesis-generating model. This framework identifies early-life vulnerability, environmental factors, and social stress as key state-level predictors that warrant further investigation. Our findings suggest a potential shift in research focus, moving from a reactive focus on adult-life risk factors toward proactive interventions targeting early-life vulnerabilities and cumulative environmental and social exposures.

## Highlights

- Recharacterizing the Predictive Landscape: This study provides new evidence that short gestation, residential radon, intimate partner violence, and occupational nickel are the key state-level predictors that merit further investigation as potentially modifiable factors of geographical disparities in respiratory failure deaths.
- A Proposed Framework for Prevention: We hypothesize an “early-life and cumulative exposure” framework that integrates siloed domains—from perinatal health to occupational safety—into a cohesive public health strategy.
- Potential Policy Priorities: Our analysis identifies several state-level predictors that, if confirmed in future research, could inform public health priorities: reducing preterm births, mandating radon testing, enforcing nickel exposure limits, and integrating IPV screening into healthcare.

## 1 Introduction

### 1.1 Traditional risk attribution and its limits

Respiratory failure, a critical end-stage manifestation of numerous diseases, remains a leading cause of global mortality, with authoritative reports consistently ranking conditions like lower respiratory tract infections and Chronic Obstructive Pulmonary Disease (COPD) among the top causes of death worldwide (1). Conventional epidemiological models have long attributed this risk primarily to behavioral and environmental factors, notably smoking and ambient air pollution, which constitute the foundation of current public health strategies (2). However, persistent geographical disparities within the United States (US.) reveal the inadequacy of this approach. Analyses have identified clusters of high lung cancer mortality in areas with low smoking prevalence, such as counties along the Mississippi River in Missouri and Western Mississippi (3). Concurrently, COPD mortality trends show a striking geographical and sex-based divergence, with rates rising among women in the US. Midwest and in nonmetropolitan areas, contrasting with general declines (4). These unresolved patterns underscore the urgent need to identify novel, state-level predictors of respiratory failure mortality beyond conventional risk factors.

### 1.2 A machine learning approach

Traditional, hypothesis-driven approaches are ill-equipped to capture these complex, high-dimensional interactions. This limitation necessitates methods like machine learning (ML). ML is uniquely suited for this task, as it can identify nonlinear relationships and interactions from large datasets without prior assumptions (5). To address the “black box” challenge, we integrate the Shapley Additive exPlanations (SHAP) framework, which provides consistent, quantitative explanations for model predictions (6). This combination of data-driven discovery and rigorous interpretability enables objective identification of key predictors.

### 1.3 Study objectives

Based on the aforementioned background, this study aims to leverage US. state-level population health data to achieve the following three core objectives in order to address a critical gap in understanding the predictors of respiratory mortality disparities:

(a) To develop, compare, and validate an optimal ML model (comprehensively evaluated by R-squared (R^2^), Root Mean Square Error (RMSE), Mean Absolute Error (MAE), and generalization diagnostics) for predicting state-level, age-adjusted respiratory failure mortality. (b) Unbiasedly identify key risk factors using SHAP, moving beyond conventional approaches. (c) Elucidate the potential multi-level hypothesized pathways of the identified predictors, drawing on existing literature.

## 2 Methods

### 2.1 Data sources and preprocessing

This study utilized U.S. state-level data selected for their policy relevance and alignment with the research objectives. Age-adjusted mortality rates for respiratory failure (ICD-10: J96.-Respiratory failure, not elsewhere classified) from 2018 to 2021 were obtained from the Centers for Disease Control and Prevention Wide-ranging Online Data for Epidemiologic Research (CDC WONDER) database. Deaths with COVID-19 (ICD-10: U07.1) listed as the underlying cause were excluded from the analysis. Sixty-eight socio-environmental variables (SEVs) were sourced from the Global Burden of Disease (GBD) study. A complete list of these 68 SEVs, including their definitions, measurement units, and categorization, is provided in Supplementary Table 1. The variables spanned three domains: early-life and reproductive health (e.g., short gestation, low birth weight), environmental exposures (e.g., residential radon, occupational nickel, ambient PM_2.5_), and social/psychosocial stressors (e.g., intimate partner violence, bullying victimization, poverty). The datasets were merged by state and year to form a consolidated analytical dataset. To ensure data integrity, the integrated dataset underwent rigorous preprocessing. For numerical features, the missing data mechanism was first examined and assumed to be missing at random (MAR). Given the low proportion of missing values (< 2%), mean imputation was applied. Furthermore, outliers—defined as observations beyond 1.5 times the interquartile range (IQR)—were Winsorized to the nearest non-outlying value.

### 2.2 Feature selection and model development

Given the limited sample size (*N* = 204, comprising 51 states over 4 years, with each state contributing one observation per year), this study implemented a structured modeling pipeline to ensure robust feature selection and reliable performance estimation. The underlying deaths per state-year ranged from 10 to 1,238, and state population from approximately 600,000 to 39 million. The dataset was partitioned using a stratified sampling approach that preserved temporal structure, with 70% of observations from each year allocated to training and the remaining 30% reserved as an independent test set. This strategy maintained temporal representation in both splits while preventing data leakage across years. The modeling pipeline proceeded in three sequential phases to ensure complete data isolation between development and evaluation:

- Feature Selection: Recursive feature elimination (RFE) with cross-validation was applied exclusively to the training data to identify an optimal feature subset. Through systematic evaluation, retaining 25 features demonstrated the optimal balance between model complexity and predictive performance, as reducing features to 20 resulted in a notable 15% increase in cross-validated RMSE (Supplemental Figure 1).
- Hyperparameter Optimization: Model hyperparameters were subsequently tuned through exhaustive grid search using 10-fold cross-validation within the training set, with RMSE minimization as the objective function. Because the dataset contains repeated observations from the same state across multiple years, an ideal approach would be state-grouped cross-validation (e.g., GroupKFold or leave-one-state-out) to prevent data leakage. However, given the limited sample size (*N* = 204, with only 4 years per state), grouped validation would leave out only 4 observations per fold, making the training set too small for stable estimation. Therefore, we adopted standard random-shuffle cross-validation while acknowledging that this may yield slightly optimistic performance estimates. To partially mitigate temporal leakage, we performed stratified sampling by year when splitting the training and test sets (70%/30%), ensuring that each year contributed proportionally to both splits.
- Final Evaluation: The generalization capability of optimally configured models was rigorously assessed on the completely withheld test set that had no involvement in either feature selection or parameter tuning.

This dual-validation framework—combining cross-validation for model development with strict hold-out testing for final evaluation—was applied across twelve machine learning algorithms spanning diverse methodological families: linear models (Linear Regression, Bayesian Ridge), regularized linear models (ElasticNet, ElasticNetCV, LinearSVR), matching pursuit [Orthogonal Matching Pursuit (OMP)], tree-based bagging (Random Forest), boosting (Gradient Boosting Machine (GBM), XGBoost, AdaBoost, CatBoost), with a single decision tree as a baseline.

The final GBM model was optimized using grid search with 10-fold cross-validation on the training set. The chosen hyperparameters are: n_estimators = 100, learning_rate = 0.1, max_depth = 3, min_samples_split = 2, min_samples_leaf = 1, subsample = 1.0, max_features = None, loss = ‘squared_error', and random_state = 42. A complete list of hyperparameters is provided in Supplementary Table 2. The analysis code is available from the corresponding author upon request.

### 2.3 Model performance evaluation

Model performance was rigorously evaluated on a hold-out test set using six complementary metrics: RMSE, MAE, R^2^, median absolute error (MedAE), mean absolute percentage error (MAPE), and explained variance score (EVS). This multi-faceted approach assessed different aspects of prediction quality, from error magnitude to variance explanation.

To compare prediction stability across models, we analyzed the distribution of prediction errors using boxplots. This visualization revealed critical differences in model robustness, including error spread, central tendency, and frequency of extreme outliers. The best-performing model underwent additional diagnostic checks. Residual analysis verified model assumptions and fit, while learning curves assessed data efficiency and potential overfitting.

### 2.4 Model interpretability analysis using SHAP

To transform the optimal model from a “black box” into a transparent and actionable tool, we conducted a multi-level interpretability analysis using the SHAP framework. This analysis was designed to uncover the predictors at both the population and individual levels.

- Global Feature Importance: A SHAP summary plot was generated to rank features by their mean absolute SHAP values, visualizing both the magnitude and direction (positive/negative) of each feature's impact on the model output across the dataset.
- Feature Interaction Analysis: SHAP dependence plots were employed to examine the relationship between individual feature values and their corresponding SHAP values. Points in these plots were colored by the values of a second, potentially interacting feature to visualize non-linear relationships and interaction effects.
- Local Interpretation: For individual predictions, SHAP force plots were used to decompose the model's output, illustrating how each feature value contributed to shifting the prediction from the baseline value.

## 3 Results

### 3.1 Descriptive statistics and spatiotemporal patterns

**Figure 1:**
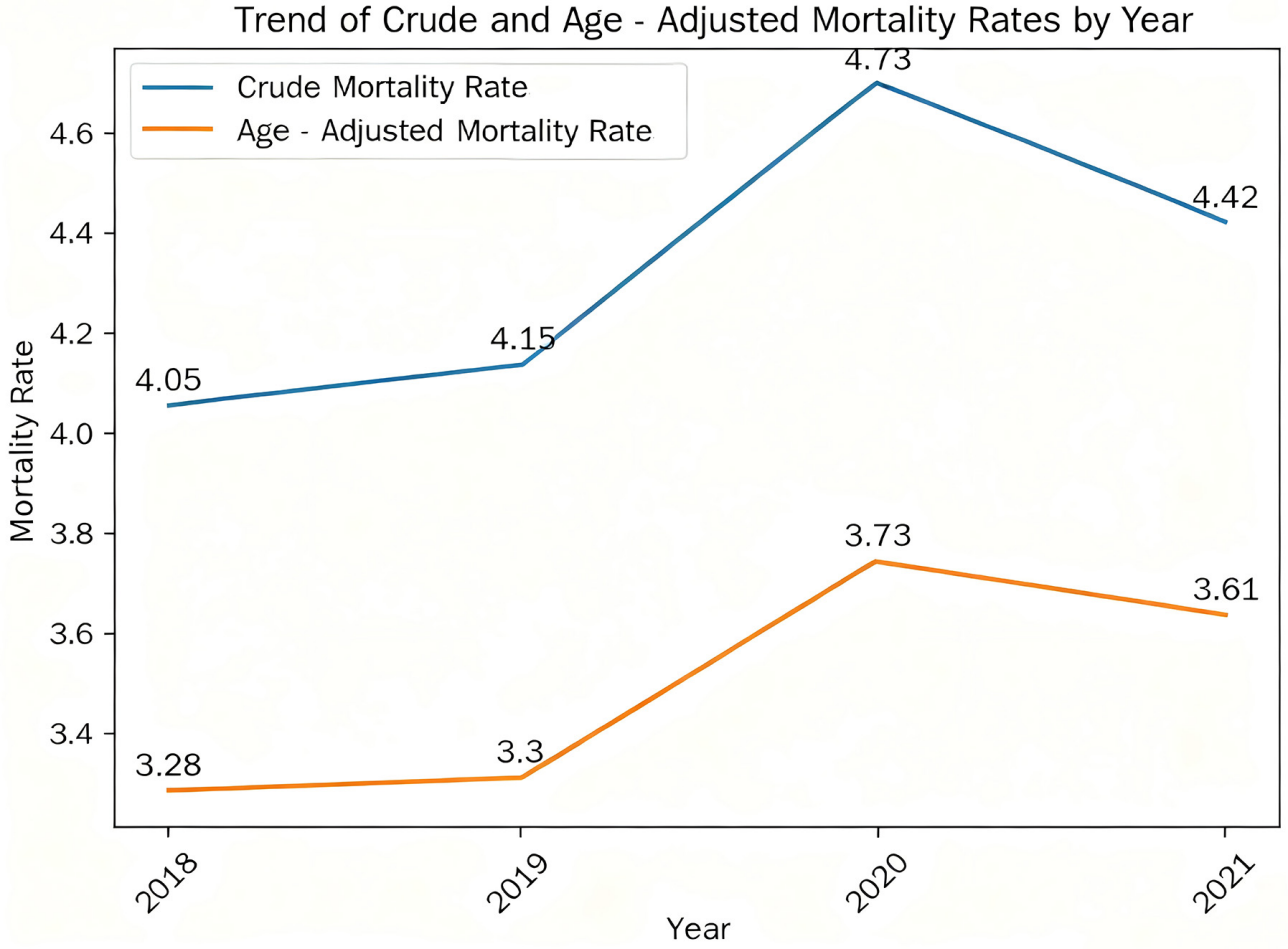
Trends in crude and age-adjusted respiratory failure mortality rates, United States, 2018–2021 (mortality rates per 100,000).

**Figure 2:**
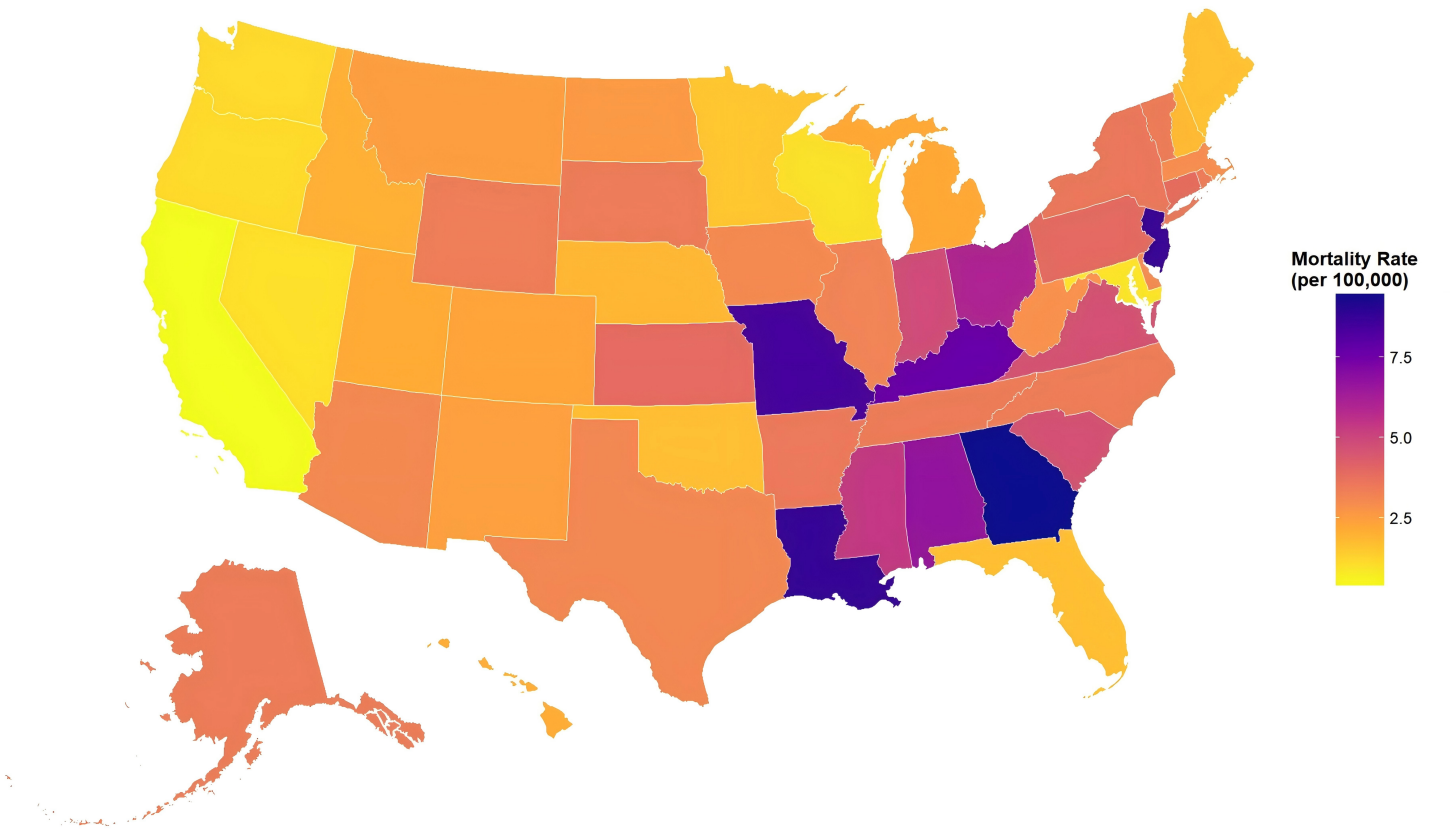
Geographic variation in age-adjusted respiratory failure mortality across U.S. States, 2018–2021 (Darker colors indicate higher mortality rates).

The final dataset included 204 annual observations across 51 US. states (including Washington D.C.) from 2018 to 2021. Analysis revealed significant fluctuations in respiratory failure mortality during the study period. As shown in Figure 1, both crude and age-adjusted mortality rates followed a similar trajectory: increasing from 2018 (4.05 and 3.28 per 100,000, respectively) to 2019, rising sharply in 2020 (peak of 4.73 and 3.73), and declining in 2021 (4.42 and 3.61). This pattern suggests a notable impact from the COVID-19 pandemic in 2020, highlighting the vulnerability of the healthcare system and the population's respiratory health to a major shock, with partial recovery the following year. Although COVID-19 deaths are coded separately, the pandemic may have indirectly increased respiratory failure mortality through healthcare system strain and delayed care for chronic conditions. Considerable interstate variation was observed in age-adjusted mortality (mean 3.44, SD 2.30), with distinct geographical clustering apparent in the spatial distribution map (Figure 2). High-risk areas, such as the “Tobacco Belt,” were clearly identified, providing critical context for subsequent analyses of state-level predictors.

### 3.2 Model performance comparison and optimal model selection

**Figure 3:**
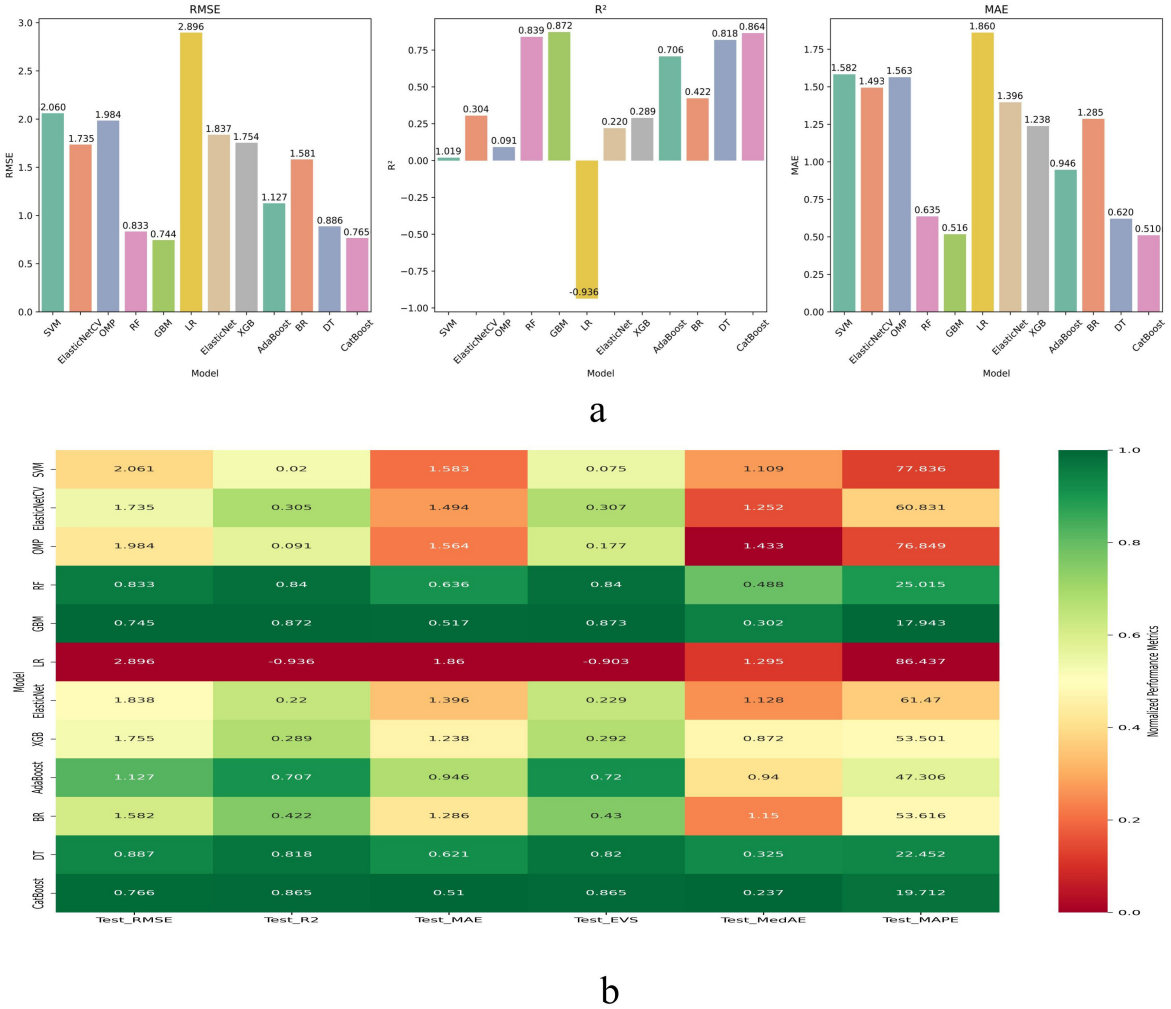
**(a)** Comparison of RMSE, R^2^, and MAE of 12 Machine Learning Models on the Training Set; **(b)** Heatmap Comparison of Multi-Metric (RMSE, R^2^, MAE, EVS, MedAE, MAPE) Performance of Different Machine Learning Models on the Test Set. The dataset was split at 70% training / 30% test with stratified temporal preservation; model tuning used 10-fold cross-validation on the training set only to avoid data leakage.

Based on the comprehensive evaluation across training and test sets, the GBM model demonstrated superior and well-balanced predictive performance. As shown in Figure 3a, the GBM model achieved highly competitive predictive accuracy on the independent test set, with a Test_RMSE of 0.744, a Test_MAE of 0.516, and a high Test_R^2^ of 0.872. This combination of low error metrics and high explanatory power on unseen data indicates that the model effectively captures the underlying mortality trends without substantial overfitting.

The multi-metric heatmap comparison (Figure 3b) confirmed GBM's robust and balanced performance. It ranked at the top for most key test-set metrics, including RMSE, R^2^, EVS, and MAPE. Although CatBoost showed marginally better performance on MAE and MedAE, GBM remained consistently strong across the broader set of metrics. Quantitatively, GBM achieved a slightly lower test RMSE than CatBoost (0.745 vs. 0.766) and a higher test R^2^ (0.872 vs. 0.865). GBM also outperformed CatBoost on MAPE (17.94% vs. 19.71%) and EVS (0.873 vs. 0.865), while CatBoost had marginally lower MAE (0.510 vs. 0.517) and MedAE (0.237 vs. 0.302). Given that no single model dominated all metrics, we selected GBM for its overall balanced performance and its greater model maturity, which facilitates reproducibility and SHAP interpretation. The differences between GBM and CatBoost were small relative to the overall prediction error, and the choice does not affect the main conclusions about feature importance, as the top four predictors were highly consistent across top-performing models.

**Figure 4:**
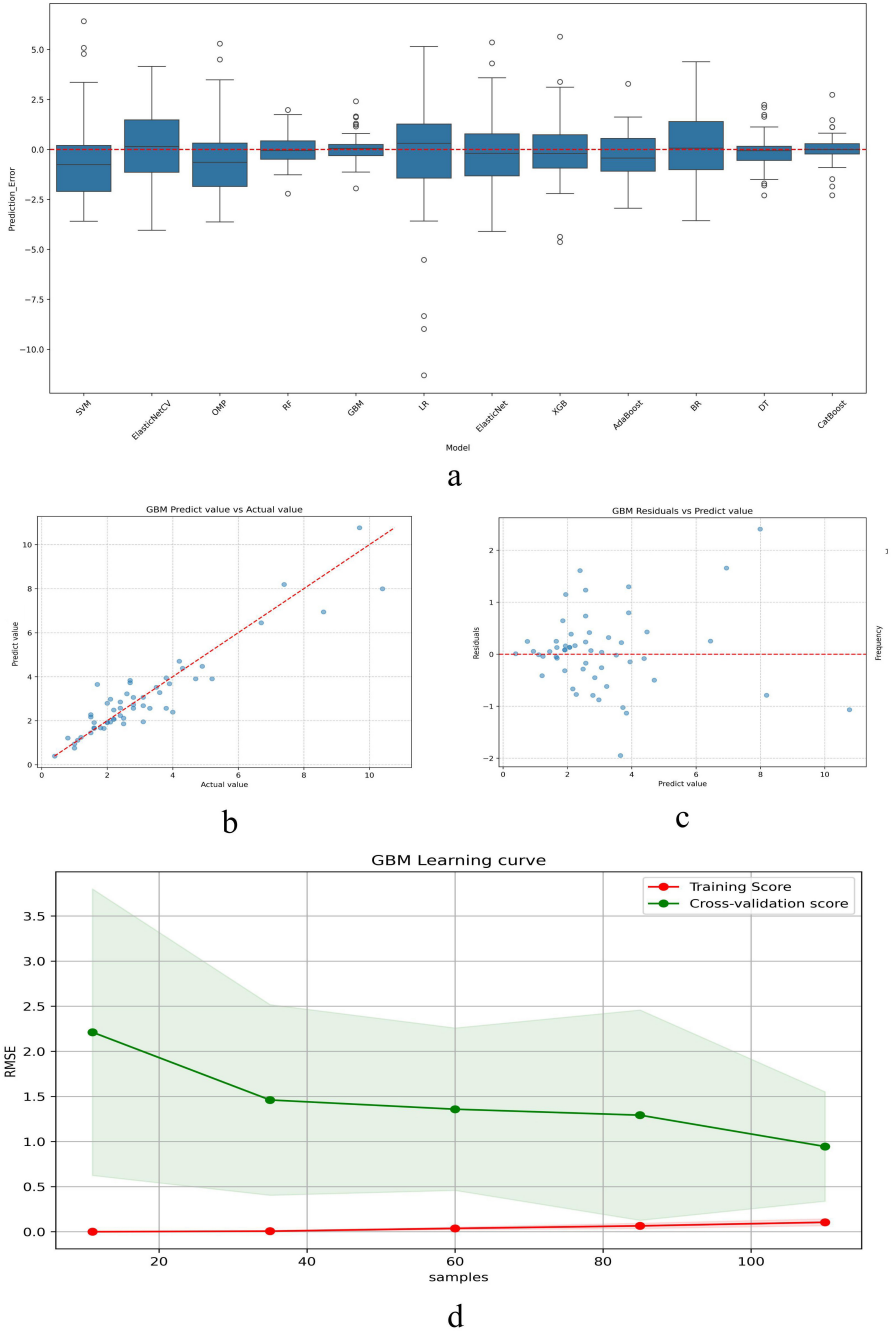
**(a)** Box plot of prediction errors for the evaluated models; **(b)** Scatter plot of predicted versus actual values for the GBM model; **(c)** Residual plot of the GBM model; **(d)** Learning curves of the GBM model. Model validation included residual diagnostics and learning curve analysis to assess overfitting and generalization.

Further analysis of prediction errors confirmed the model's robustness, with GBM exhibiting the tightest error distribution and a median closest to zero among all models evaluated (Figure 4a).

To comprehensively evaluate the GBM model's fitting performance and generalization capability, we conducted residual analysis and learning curve diagnostics. As shown in Figure 4b, predicted versus actual values were closely distributed along the diagonal, indicating high overall accuracy. Figure 4c reveals that residuals were randomly and uniformly scattered around zero without evident trends or heteroscedasticity, suggesting no systematic bias and adequate capture of underlying data patterns. Furthermore, the learning curves (Figure 4d) showed that training and validation errors converged synchronously as sample size increased, with a consistently small generalization gap, confirming strong generalization ability without overfitting.

Collectively, these results validate GBM as a well-specified, robust, and reliable model, justifying its selection for subsequent predictor analysis.

### 3.3 Interpretability analysis results

To elucidate the decision-making mechanism of the GBM model, we conducted a multi-dimensional interpretability analysis using the SHAP framework.

#### 3.3.1 Global feature importance

**Figure 5:**
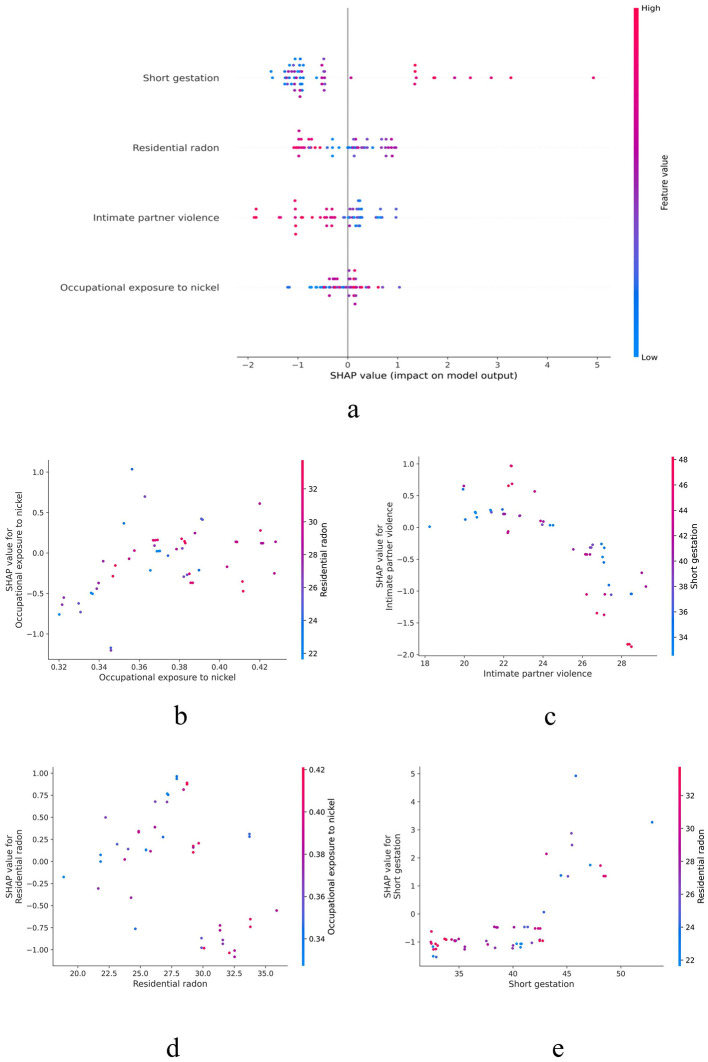
**(a)** Beeswarm Plot of SHAP Value Distribution; **(b-e)** SHAP dependence plots showing nonlinear patterns in model predictions. These patterns should be interpreted as hypothesis-generating, not as evidence of biological synergy. Visualizations derived from the optimally tuned GBM model using held-out test data.

Global feature importance was analyzed using a beeswarm plot (Figure 5a), which displays only the top four features based on SHAP importance. These core predictors, short gestation, residential radon, intimate partner violence (IPV), and occupational nickel exposure, exhibited substantially higher SHAP values than other variables, confirming their status as the most critical predictors, which collectively account for the primary share of the model's explanatory power.

#### 3.3.2 Feature effects and patterns

SHAP dependence plots revealed exploratory visual patterns suggesting that the model's predictions vary with combinations of features (Figures 5b–e). Specifically, states with higher levels of both occupational nickel exposure and residential radon showed model predictions that exceeded what would be expected from the sum of their individual contributions, a possible non-linear co-patterning that warrants formal interaction testing in future studies. Additionally, the association of short gestation with predicted mortality appeared to depend on residential radon levels, with the direction of the SHAP values varying across the range of radon exposure. A similarly complex pattern was observed for IPV, whose SHAP values varied with short gestation prevalence. We emphasize that these SHAP-based visualizations are exploratory and hypothesis-generating; formal statistical interaction tests (e.g., including product terms in regression models, calculating interaction SHAP values, or partial dependence/ICE curves) are needed to confirm whether genuine interactions exist.

These findings illustrate that in our model, early-life, social stress, and environmental factors jointly predict state-level mortality. The observed nonlinear and interactive patterns generate hypotheses about potential pathways that warrant investigation in individual-level cohort studies, underscoring the need to formally test for potential multi-dimensional interactions in future etiologic or individual-level research.

#### 3.3.3 Visualization of decision logic

**Figure 6:**
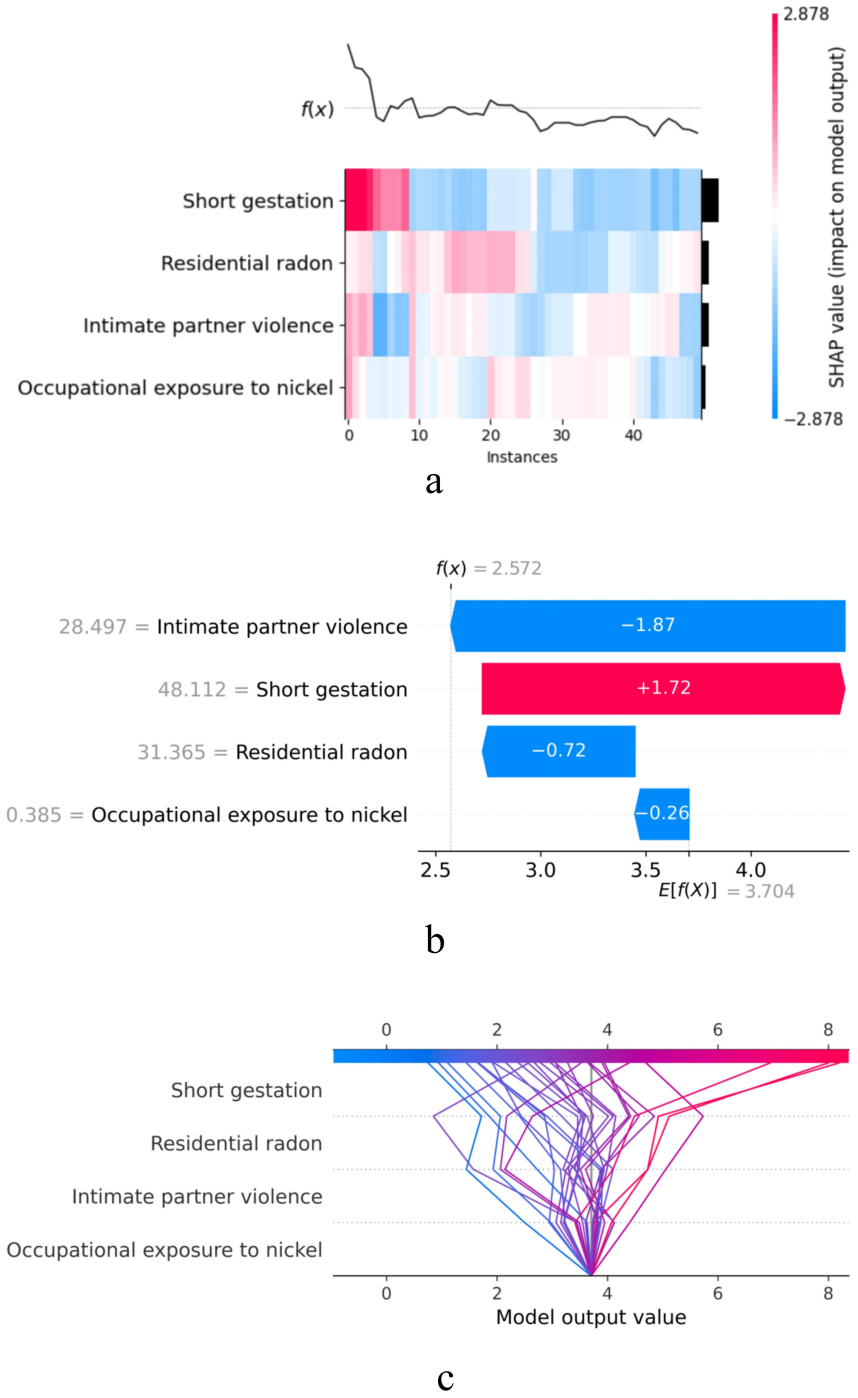
**(a)** SHAP heatmap with cluster analysis of feature contributions; **(b)** Deconstruction of individual predictions using SHAP waterfall plots for two representative samples; **(c)** Comparison of feature contribution paths across different samples using SHAP decision plots. Interpretations are limited to predictive patterns within the state-level ecological framework.

Through heatmaps, waterfall plots, and decision plots, we further decoded the GBM's prediction mechanism. Cluster analysis via SHAP heatmap (Figure 6a) identified distinct state groupings driven by different feature configurations, some dominated by high short gestation with low radon, others by the opposite pattern, revealing substantial population heterogeneity. The waterfall plot (Figure 6b) deconstructed individual predictions from the baseline (E[f(X)] = 3.704) to final output, illustrating how features exerted push-pull effects on mortality estimates. Decision plots (Figure 6c) visually compared cumulative feature contributions across states, showing how specific combinations (e.g., high IPV with high nickel exposure) substantially elevated predicted risk.

Together, these SHAP-based visualizations illustrate how the model uses early-life, environmental, and social stress factors to predict spatial disparities in respiratory failure mortality. These exploratory results generate hypotheses for future public health research.

## 4 Discussion

### 4.1 Key predictors and a proposed hypothesis-generating framework

Beyond achieving high predictive accuracy (GBM, R^2^ = 0.872), our study identifies a novel set of state-level predictors of respiratory failure mortality: short gestation, residential radon, IPV, and occupational nickel exposure. These findings diverge from the prevailing approach, which focuses on immediate respiratory insults such as smoking or ambient PM_2.5_.

Importantly, our ecological design precludes causal inference at the individual level. The high predictive importance of a variable in our model does not demonstrate that it is a causal driver of respiratory failure; rather, it indicates a strong state-year association that may reflect direct effects, indirect effects, confounding, or a combination thereof. The following discussion therefore interprets our findings as hypothesis-generating, proposing potential pathways drawn from existing individual-level literature that could explain the observed ecological associations, should future research confirm them.

The absence of these traditional factors as top-rank predictors in our model should be interpreted with caution. Several methodological reasons may explain this finding, none of which challenges the established health effects of smoking or PM_2.5_ at the individual level. First, limited state-level variation in smoking rates may reduce its discriminatory power in an ecological model. Second, collinearity with gestational and other variables could lead the algorithm to select correlated alternatives. Third, the modest sample size (*N* = 204) may introduce instability in feature ranking. Fourth, although our *post-hoc* analysis revealed a moderate ecological correlation between smoking and short gestation (*r* = 0.42, *p* < 0.001), this co-pattern is exploratory and hypothesis-generating; it does not constitute evidence of mediation, and any such interpretation remains highly speculative without individual-level longitudinal data. Fifth, residential radon's strong state-level heterogeneity may enhance its detectability, whereas the more uniformly distributed ambient PM_2.5_ could be less discriminative. None of these observations invalidate traditional risk factors; rather, they highlight the importance of study design in predictive modeling.

Based on these findings, we propose a hypothesis-generating framework, tentatively termed the “early-life and cumulative exposure” framework, to guide future research on the life course transitions that might lead to respiratory failure (7). This framework suggests, as a testable hypothesis, that the risk of respiratory failure accumulates over a lifetime, originating from early-life physiological vulnerabilities and prolonged exposures across residential, occupational, and psychosocial domains.

#### 4.1.1 Lifecourse vulnerability: short gestation

The strong predictive importance of short gestation in our state-level model is consistent with, but does not directly test, the Developmental Origins of Health and Disease (DOHaD) hypothesis (8). Preterm birth critically interrupts the alveolar stage of lung development, which can lead to bronchopulmonary dysplasia (BPD) (9). Importantly, even in the absence of BPD, individuals born extremely preterm commonly exhibit abnormal lung function and a higher risk of childhood wheeze and asthma, a condition now recognized as post-prematurity respiratory disease (PPRD), which is associated with early-onset adult lung disease and subsequent reductions in quality-adjusted life years (10).

The underlying pathological sequelae encompass a spectrum of impairments. These include varying degrees of: impaired alveolarization; persistent inflammation; abnormal vasculature and vascular remodeling; and airway alterations, which may result in tracheobronchomalacia (9). Furthermore, emerging evidence suggests this early-life vulnerability may be maintained through epigenetic modifications [e.g., DNA methylation in genes regulating inflammation and lung development (11)], which create a pro-inflammatory phenotype that increases susceptibility to COPD later in life (12). This hypothesized pathway requires direct testing in individual-level longitudinal studies that track preterm birth and subsequent respiratory outcomes.

Our ecological finding suggests that state-level variation in preterm birth rates may be a meaningful predictor of respiratory failure mortality, generating the testable hypothesis that interventions to reduce preterm birth could have population-level respiratory benefits.

A note on potential confounding: The strong predictive importance of short gestation in our model raises the question of whether this reflects a direct biological effect of preterm birth on later respiratory health, or whether short gestation serves as a proxy for unmeasured socioeconomic disadvantages that also increase respiratory failure risk. Existing individual-level evidence supports both possibilities: preterm birth directly impairs lung development (as discussed above), but preterm birth is also more common among socioeconomically disadvantaged populations, which face multiple other respiratory hazards (e.g., substandard housing, poor nutrition, chronic stress). Our state-level data cannot disentangle these pathways. Future individual-level studies with detailed socioeconomic and biological measurements are needed to determine whether short gestation has an independent causal effect on respiratory failure mortality or whether it primarily reflects the cumulative burden of social disadvantage.

#### 4.1.2 Generating hypotheses about environmental and occupational exposures: cumulative threats

The prominence of residential radon and occupational nickel exposure as state-level predictors reveals ecological patterns that warrant further investigation. Existing individual-level literature has documented plausible pathways for both exposures.

For residential radon, published mechanistic studies have shown that inhalation of its decay products (which emit alpha particles) can cause DNA damage, chronic inflammation, and oxidative stress in lung tissue-ultimately leading to fibrosis and impaired gas exchange (13). This risk has been demonstrated to be cumulative, driven significantly by both the duration and intensity of exposure (14). The predictive power of radon in our model is underscored by its highly heterogeneous distribution across the US, where high-resolution mapping reveals over 83 million people reside in homes exceeding the action level, many in traditionally “low-risk” zones (15). This persistent threat is potentially exacerbated by climate change impacts on radon migration and the paradoxical effect of energy-efficient buildings that can increase indoor accumulation (16). The observed higher burden in males in prior studies further highlights the need for targeted public health strategies (17).

For occupational nickel exposure, experimental models have demonstrated a progression from acute lung inflammation and injury to chronic fibrosis following nickel inhalation (18), with evidence of persistent neutrophilic infiltration, oxidative stress, and fibrotic remodeling (19). Epidemiological studies have documented associations between cumulative nickel exposure and increased risk of COPD and asthma—both critical precursors to respiratory failure (20, 21). Our ecological finding that state-level nickel exposure predicts respiratory failure mortality generates the hypothesis that chronic nickel exposure may represent a persistent and progressive insult to respiratory health. Documented exposures in sectors such as welding, metal manufacturing, and barbershops (22, 23) suggest potentially modifiable sources if causal relationships are confirmed.

In summary, both radon and nickel exposure represent plausible environmental determinants of respiratory health based on existing literature. Our ecological associations generate specific, testable hypotheses that should be evaluated in individual-level cohort studies with detailed exposure assessment.

#### 4.1.3 Generating hypotheses about embodied social stress: intimate partner violence

The robust association between IPV and respiratory failure mortality in our state-level model is consistent with, but does not directly demonstrate, potential pathways from psychosocial stress to respiratory health. Existing individual-level literature has proposed several mechanisms that could explain such an association.

First, physiological pathways: Published research has shown that chronic stress exposure can induce persistent activation of the hypothalamic-pituitary-adrenal (HPA) axis, potentially leading to glucocorticoid receptor resistance and subsequent systemic chronic inflammation characterized by elevated IL-6, TNF-α, and CRP levels (24). Such a pro-inflammatory state could, in principle, exacerbate airway inflammation and impair lung tissue repair capacity (25).

Second, behavioral pathways: Studies have documented that IPV survivors show elevated rates of health-risk behaviors, such as smoking (often used as a coping mechanism), and poor medication adherence due to economic control and social isolation (26, 27).

Our ecological finding of a strong state-level association between IPV prevalence and respiratory failure mortality generates the hypothesis that these individual-level pathways, physiological, behavioral, or both, may operate at the population level. Specifically, we hypothesize that states with higher IPV prevalence may have higher respiratory failure mortality because IPV could contribute to chronic inflammation, health-risk behaviors, or barriers to healthcare access. This hypothesis requires testing in individual-level prospective studies that measure IPV exposure, potential mediating pathways, and respiratory outcomes.

### 4.2 Implications for public health

The following policy implications assume that the state-level predictive associations we observed reflect causal relationships at the individual level-an assumption that requires empirical validation. These recommendations should therefore be interpreted as hypothetical priorities for future effectiveness research.

If substantiated by future individual-level research, our findings would support a reorientation of public health policy. we suggest moving beyond a reactive, adult-focused model and toward a proactive, lifecourse-oriented, and multi-sectoral strategy. To directly address the key predictors identified in this study, we propose the following immediate actions:

- Strengthening foundational health: Federal and state maternal and child healthprograms should set specific targets to reduce preterm birth rates by 5–10%. This can be achieved by expanding proven prenatal interventions, including enhanced nutritional support (e.g., WIC) and smoking cessation programs.
- Ensuring healthy environments: If causal relationships are confirmed by future research, policymakers could consider mandating radon testing in homes, potentially supported by remediation subsidies whose optimal level would require further cost-effectiveness analysis. Concurrently, occupational safety regulators should consider establishing and enforcing stricter exposure limits for toxicants like nickel.
- Integrating health and social services: Implement routine IPV screening in primary and pulmonary care, supported by standardized tools, provider training, and clear referral pathways to social and legal services.

If the observed associations are confirmed causally, such a prevention network could help address the complex risk profile identified by our analysis.

### 4.3 Limitations and future directions

A central limitation of this study is its ecological design. The high predictive accuracy of our GBM model (R^2^ = 0.872) indicates that the selected features are strongly associated with state-level mortality. However, predictive importance does not equal causal importance. A variable may be a strong predictor because it is a proxy for an unmeasured confounder, because it lies on a causal pathway, or because of reverse causation. Our SHAP analysis identifies which variables the model “learned” to rely on for prediction, not which variables are causally responsible for mortality. Readers should interpret our “key predictors” language throughout as referring to predictive importance within this specific model, not as causal claims. Additional methodological limitations are as follows: First, the socio-environmental variables from the GBD study, while rigorous, were not externally validated; employing complementary US. data sources (e.g., BRFSS, American Community Survey) in future work would enhance validity. Second, residual confounding and potential inconsistencies in mortality coding may influence the results. Third, the modest sample size (*N* = 204 state-year observations) may lead to instability in feature ranking and overfitting. Fourth, our cross-validation strategy did not explicitly group observations by state. Because the same state appears in multiple years, random shuffling of folds may lead to data leakage (i.e., observations from the same state could appear in both training and validation folds), potentially resulting in overoptimistic performance estimates. Fifth, we did not report uncertainty estimates (e.g., confidence intervals or standard deviations) for performance metrics such as RMSE or SHAP values, due to the computational cost of repeated model fitting with 12 algorithms and 10-fold CV. Future studies should provide bootstrap or repeated cross-validation estimates to better quantify prediction stability. Future studies with larger samples (e.g., more years per state or county-level data) should also adopt state-grouped cross-validation (e.g., GroupKFold or leave-one-state-out) to obtain more conservative generalization estimates.

Rather than undermining the study's value, these limitations provide a clear roadmap for future scientific inquiry. The primary contribution of this work is the generation of novel, compelling hypotheses that challenge conventional approaches. Future research should prioritize:

- Individual-level validation: Employ prospective cohort studies to establish longitudinal associations and causal relationships between the identified factors (e.g., short gestation, IPV) and individual respiratory failure risk.
- Hypothesis testing of proposed pathways: Investigate whether the pathways suggested by existing literature, such as the influence of preterm birth on adult lung function or the role of chronic psychosocial stress in lung inflammation, are supported in individual-level data, rather than assumed from ecological patterns.
- Intervention development and evaluation: The public health actions recommended above must be formally evaluated. Future work should assess the efficacy and cost-effectiveness of these specific strategies, such as the impact of radon subsidy programs on exposure reduction or the health outcomes of integrated IPV screening in clinical settings.

In summary, while acknowledging its limitations, this study's primary contribution lies in generating transformative hypotheses. The research trajectory outlined here, validating associations, testing hypothesized pathways, and evaluating interventions, provides a concrete agenda to advance our understanding of respiratory failure mortality.

## 5 Conclusion

This study reveals that the state-level predictors of respiratory failure mortality are more complex than traditionally recognized. Our findings identify novel associations that challenge conventional approaches and generate specific hypotheses about potential early-life, environmental, and social determinants, hypotheses that now require rigorous testing in individual-level prospective studies. Our findings suggest that the most significant state-level predictors are embedded in early life, the residential and occupational environment, and social contexts. By offering a different perspective from conventional approaches, this research provides hypotheses and exploratory findings that may help guide future research toward a more comprehensive public health strategy against respiratory failure.

## Data Availability

The datasets presented in this study can be found in online repositories. The names of the repository/repositories and accession number(s) can be found in the article/Supplementary material.
