# Supplementary figures and images for: Identifying early-life, environmental, and social stressors as key predictors of U.S. respiratory failure mortality: a machine learning study
Source: Front Public Health. 2026 Jun 26;14:1839753. doi: 10.3389/fpubh.2026.1839753 (PMC13350441; doi:10.3389/fpubh.2026.1839753)

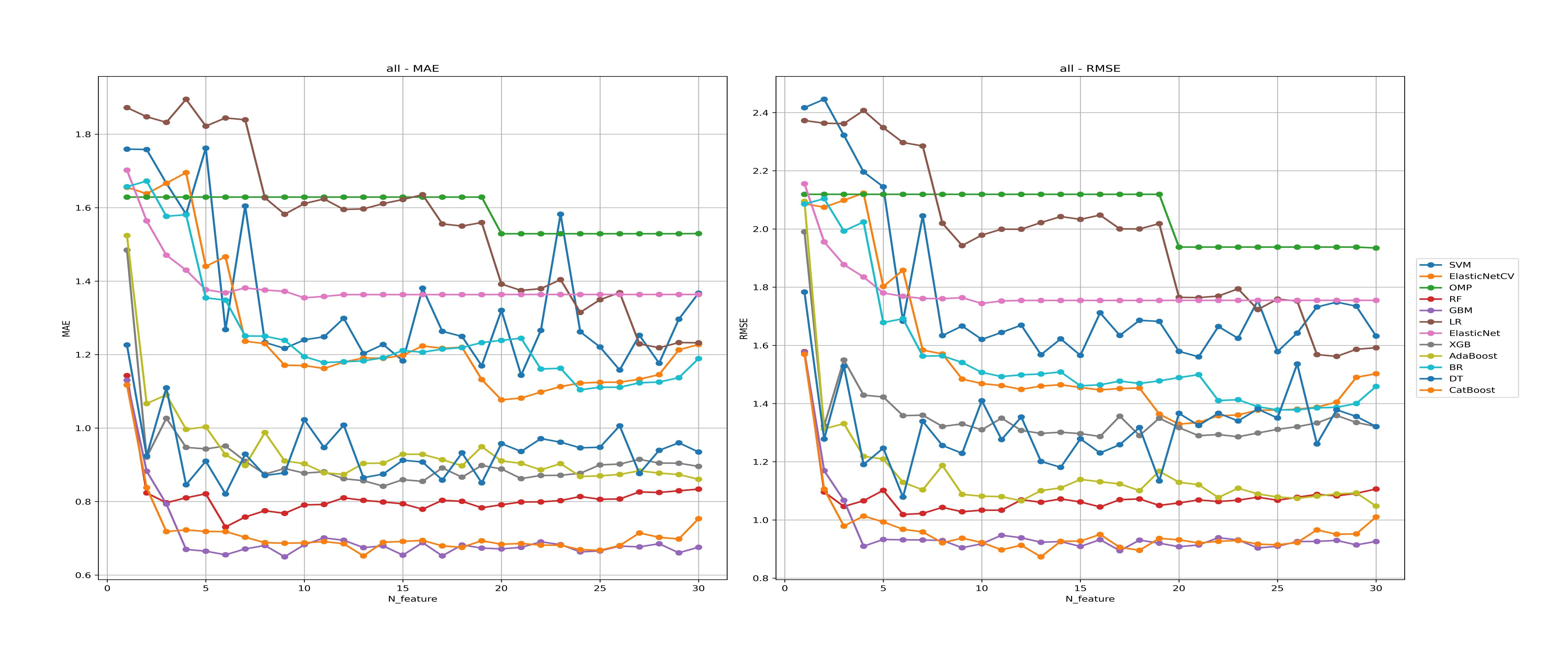

Supplement: Supplementary file 3 [file Image_1.jpeg]
